# The Association between the Dietary Antioxidant Index and Weight Status in Primary School Students: An Epidemiological Study

**DOI:** 10.3390/nu16111667

**Published:** 2024-05-29

**Authors:** Stamatia Kokkou, Venetia Notara, Aikaterini Kanellopoulou, George Antonogeorgos, Andrea Paola Rojas-Gil, Ekaterina Kornilaki, Areti Lagiou, Demosthenes Panagiotakos

**Affiliations:** 1 Laboratory of Hygiene and Epidemiology, Department of Public and Community Health, School of Public Health, University of West Attica, Alexandras Avenue 196, 115 21 Athens, Greece; skokkou@uniwa.gr (S.K.); vnotara@uniwa.gr (V.N.); alagiou@uniwa.gr (A.L.); 2 Department of Nutrition and Dietetics, School of Health Sciences and Education, Harokopio University, Thiseos 70, 176 76 Athens, Greece; katerkane@gmail.com (A.K.); gantonogeorgos@gmail.com (G.A.); 3 Laboratory of Basic Health Sciences, Department of Nursing, Faculty of Health Sciences, University of Peloponnese, Karaiskaki 70, 221 00 Tripoli, Greece; apaola71@yahoo.com.mx; 4 Department of Preschool Education, School of Education, University of Crete, 741 00 Gallos, Greece; ekornilaki@edc.uoc.gr

**Keywords:** children, overweight/obesity, DAI, dietary antioxidant index, antioxidants, cardiovascular disease

## Abstract

Obesity is an emerging threat and a current challenge for children and adolescents worldwide. The aim of the present work was to evaluate the relationship between the Dietary Antioxidant Index (DAI) and the weight status of students in early adolescence. A sample of 1580 students aged 10–12 years from 47 primary schools in Greece were enrolled. Anthropometric characteristics were assessed, and calculation of the Body Mass Index (BMI) was used to categorize students into two weight-status groups. Dietary habits and physical activity were evaluated using a self-completed questionnaire, and the DAI was calculated through derived micronutrients’ content, along with energy, macro-, and micro-nutrient intake. Crude and adjusted regression analysis showed a significant inverse association of the DAI and body weight status (Odds Ratio (OR): 0.719, 95% Confidence Interval (CI): 0.576; 0.897, and adjusted Odds Ration (aOR): 0.667, 95% CI: 0.489; 0.907). An antioxidant diet seems to play a protective role against increased body weight among students in early adolescence. Thus, dietary patterns rich in antioxidants should be promoted to facilitate healthy habits early in life, and to fight the obesity threat.

## 1. Introduction

The prevalence of increased body weight and obesity during childhood years, as well as pre- and early adolescence, is undoubtedly one of the most crucial and current public health threats. According to the most recent World Health Organization (WHO) report, over 300 million youngsters aged 5 to 19 years worldwide had elevated body weight, with overweight prevalence being almost fivefold and obesity prevalence about sevenfold higher than four decades ago [1]. The importance lies in the fact that early obesity is more likely to persist throughout a whole life. Evidence from a recent meta-analysis revealed that more than half of children living with obesity become adolescents with obesity, and about 4/5 of those remain with the condition in adulthood, with only about 10% becoming obesity-free after the age of 30 years [2]. Especially for the Greek population, this is an issue of great concern, since in terms of increased adiposity during childhood, Greece holds one of the highest ranks in Europe, even though according to a recent study, Greek children tend to be of lower body weight now than they were ten years ago [3].

Furthermore, the link between elevated body weight and oxidative stress has also been the subject of scientific interest. It is already known that adipose tissue increases oxidative stress through different pathways, such as the production of adipokines, which in turn increase circulating free radicals, with reactive oxygen species (ROS) being the main component [4,5]. Additionally, data from recent literature explains how the accumulation of excessive adipose tissue, and oxidative stress and inflammation occurrence are more than unbreakably bonded, as their intertwined nature results in each of them increasing the odds of higher manifestation rates for the others. This is a fact that ultimately leads to a greater risk of cardiovascular diseases (CVDs) [6,7,8,9]. However, it is already known that body weight is modifiable, and similarly, there is evidence to support that oxidative stress is a modifiable health risk factor as well [6]. Some of the proposed ways to reduce oxidative stress include antioxidant intake, such as specific antioxidant/prooxidant nutrient content [10] and consumption of certain antioxidant compound groups derived from plants [11]. Under the prism of dietary antioxidant intake, a recent systematic review paper highlighted the fact that higher adiposity rates seem to be associated with lower consumption of the six main antioxidant micronutrients, namely vitamins E and C, carotenoids, magnesium, selenium, and zinc [12].

The aim of the present work was to evaluate the potential relationship of dietary antioxidant intake on weight status in a sample of Greek preadolescents.

## 2. Materials and Methods

### 2.1. Study Design

This population-based, observational, cross-sectional research study was carried out in three major Greek regions during the academic years 2014–2015 and 2015–2016: the broader metropolitan area of Athens, Heraklion, the capital city of Crete Island, and three central Peloponnesian counties, specifically Kalamata, Pyrgos, and Sparta. These districts are representative of significant urban and exurban communities according to their population size. A sum of 47 primary schools were selected, with the use of random sampling, from a list provided by the Greek Ministry of Education. Ten primary schools were based in the three aforementioned cities on the Peloponnese peninsula, five were in Heraklion, and 32 were located in Greece’s capital city.

### 2.2. Participants

A total of 1728 students, aged between 10 and 12 years, were incorporated in the study sample (795 male and 933 female students). The current study included children whose eating habits were ascertained, resulting in a final sample of 1580 students from the aforementioned five regions. Between those included and excluded, there was no difference in any of the fundamental variables (age, gender, weight status, and physical activity). Across the 47 schools, participation rates varied from 95% to 100%.

### 2.3. Power Analysis

With 80% statistical power at the <5% level of significance, the current sample was adequate to assess the impact size measures’ 15% differences in the prevalence of overweight/obesity status between the study groups.

### 2.4. Assessment of Weight Status

Students’ anthropometric (weight and height) traits were assessed using established measurement tools (i.e., a scale and tape measure). The formula for calculating Body Mass Index (BMI) was weight (in kilograms) divided by height (in meters, squared). Additionally, students’ weight status was classified as either “normal” or “overweight/obesity”, according to the International Obesity Task Force (IOTF)’s age- and sex-specific BMI cut-off standards, which link BMI up to 18 years of age (16, 17, 18.5, 25, and 30 kg/m^2^) in kid centiles [13]. Students of lower-than-normal body weight were incorporated into the “normal” group due to their small percentage. For the purposes of this analysis, the two categories of higher body weight (“overweight/obesity”) were combined into one due to the relatively small number of students who were obese.

### 2.5. Lifestyle Factors’ Assessments

The participants filled out a self-reporting questionnaire in the presence of qualified field researchers and educators, with the sole intention of clearing up any misunderstandings. It contained questions about students’ socio-demographics, such as gender and age. It also included validated versions of both a Food Frequency Questionnaire (FFQ) [14] and a Physical Activity Questionnaire (IPAQ) [15] for the assessment of students’ dietary habits and exercise levels, respectively. In addition to the IPAQ, extracurricular sports activities, such as swimming, running, or daily or weekly participation in sports teams, were assessed using the question “Do you participate in sports or a team sport outside school?” with possible answers “no” or “yes”. After evaluation of the IPAQ answers and the answer regarding extracurricular sports, participants were categorized into two groups. Regarding nutrient intake, the US Department of Agriculture’s composition tables [16] and the “Composition tables for Greek foods and recipes” [17] were used to evaluate students’ eating habits in further detail. Regarding quantification of the content of macronutrients and micronutrients, the serving sizes used were the ones proposed by the US Department of Agriculture’s composition tables and the “Composition tables for Greek foods and recipes” [16,17]; calculations were based on the respective macro/micronutrients’ content. Macronutrient content (protein, carbohydrate, and lipid) was measured in grams/serving size, out of which the energy intake was calculated (in kcal); the intake of vitamin A and selenium were measured in μg/serving size, whereas vitamins C and E, magnesium, and zinc were measured in mg/serving size.

### 2.6. The Dietary Antioxidant Index

To measure the level of antioxidant intake, the Dietary Antioxidant Index (DAI) was calculated using information derived from the FFQ regarding the intake of vitamins A, C, and E, as well as magnesium, selenium, and zinc, following the approach by Wright et al. that was first suggested in 2004 [18]. The normalization of each micronutrient intake was achieved by subtracting the mean intake and dividing the result by the standard deviation (SD). The final calculation of the DAI was the sum of the normalized micronutrient intake and then, the creation of a binary variable by separation using the median (i.e., −1.17).

### 2.7. Statistical Analysis

The data are displayed as absolute and relative (%) frequencies for categorical variables, as well as with a mean value ± SD for the continuous ones. The Kolmogorov–Smirnov test was utilized to assess and validate the normality of distribution for continuous variables among the several weight-status groups, specifically the intake of vitamins A, C, and E, magnesium, zinc, and selenium. The mean values of the weight-status groups were compared using a Student’s *t*-test regarding consumption of each of the previously listed micronutrients. Linear regression analysis was performed for the continuous variable of students’ BMI, as well as logistic regression analysis for binary body-weight status, to evaluate a potential association with the DAI. The outcomes are shown as standardized B coefficients for the former, and odds ratios (OR) and associated 95% confidence intervals (95% CI) for the latter regression models. Based on the scientific literature, predominant confounding factors were age, gender, energy intake, and physical activity (Model 2), in addition to intake of the six components of the DAI (Model 3). These were considered in order to evaluate the Index’s independent potential association. The variance inflation factor and tolerance were evaluated to determine whether multicollinearity among the independent variables was present. Analyses were performed using Stata 14.0 statistical software (M. Psarros & Assoc., Sparti, Greece), setting the level of significance at 5%.

### 2.8. Bioethics

The Institute of Educational Policy, a division of the Ministry of Education and Religious Affairs, authorized the current study, which was carried out in compliance with the 1989 Declaration of Helsinki (permission code: F15/396/72005/C1). All parties associated with this study, including participants and their guardians, school administrators, and school personnel, were fully informed about the study’s objectives and methods before being asked to sign an informed consent form.

## 3. Results

Table 1 displays the basic characteristics of the students, both collectively and individually, for every weight-status group. The participants’ mean age was 11.21 ± 0.782 years, and 54.5% of them were female. Most students with increased adiposity levels were boys (53.7%), whereas girls were dominant in the normal weight group, representing 57.6% of the study sample (*p* < 0.001). Additionally, 78.7% of students reported being physically active; however, compared to their peers, those with an augmented body weight were substantially more likely to be inactive (*p* < 0.001). Mean energy intake, as well as mean intake of macronutrients (carbohydrate, protein, fat), differed between the two weight-status groups. Finally, mean intake of the six antioxidant micronutrients, from which the DAI was calculated, did not vary between groups; however, mean intakes for vitamin A and vitamin C were only marginally non-significant (*p* = 0.072 and *p* = 0.069, respectively).

Table 2 presents the characteristics of the participants between the two DAI groups (i.e., DAI < −1.17, DAI ≥ −1.17). All macronutrient and antioxidant micronutrient intakes were significantly lower in the DAI < −1.17 group (all *p* < 0.001). However, besides the fact that mean energy intake was shown to be higher in the DAI ≥ −1.17 group (*p* < 0.001), students of the corresponding group had a significantly lower BMI (*p* = 0.004).

Linear regression analysis was performed for the possible relationship between the DAI and students’ BMI (Table 3). The DAI was inversely associated with BMI in an unadjusted analysis (*p* = 0.004); this remained significant in the full models that were adjusted for age, sex, mean energy intake, and physical activity (*p* = 0.004), and for the intakes of vitamin A, vitamin C, vitamin E, zinc, selenium, and magnesium (*p* = 0.032).

Table 4 shows the results of the logistic regression analyses for the potential association of DAI on students’ overweight/obesity status. A crude analysis showed a significant inverse association between the DAI and body weight status (OR: 0.719, 95% CI: 0.576; 0.897), which remained significant even after adjustments made in Model 2 (aOR: 0.662, 95% CI: 0.502; 0.873) and Model 3 (aOR: 0.666, 95% CI: 0.489; 0.907).

## 4. Discussion

The aim of the present study was to assess the potential association of antioxidant micronutrient consumption, through use of the Dietary Antioxidant Index (DAI), on weight status on a sample of students aged 10–12 years. Our results suggested that students who had higher DAI values were 28% less likely to have an elevated body weight than those who had lower DAI values. After adjustments for age, gender, mean energy intake, and physical activity, students who had higher DAI values were 33% less likely to have an elevated body weight than those who had lower DAI values.

To the best of our knowledge, this is one of the first studies aiming to examine this relationship. A recent study [19] evaluated the relationship between a variation of the DAI, which included manganese instead of magnesium [20], and the BMI of a sample of 593 adolescent boys aged 12–16 years. The results indicated that there was a beneficial association of higher antioxidant intakes, expressed as more elevated scores of the DAI, on the sample’s weight status [19]. Furthermore, another study on 203 adolescents living with an overweight/obesity status indicated that those with lower intakes of dietary antioxidants had a higher possibility of elevated body weight occurrence and impaired metabolic health [21]. However, a previous work, using the dietary antioxidant quality score, which excluded magnesium, on a sample of 4270 participants aged 6–18 years, showed that children and adolescents of a higher body weight had more notable intakes of some dietary antioxidants than their peers [22]. Finally, a study in Spain evaluated total antioxidant capacity, which diverged from the DAI in terms of zinc and selenium intake, on the risk of developing attributes associated with obesity in 369 children and adolescents, and concluded that there was a statistically significant inverse association but only in participants with an elevated body weight [23].

There is strong scientific evidence indicating a beneficial role of antioxidant diets on oxidative stress and the prevention of CVDs. A recent analysis based on data of the National Health and Nutrition Examination Survey (NHANES) showed that higher scores of the DAI were associated with decreased all-cause mortality, as well as mortality from CVDs, in adults affected by type II diabetes mellitus [24]. Additionally, a study using the manganese variation of the DAI concluded that higher scores of the index were associated with increased levels of serum anti-inflammatory and antioxidant components, thus indicating better protection against oxidative stress [25]. Another study from Poland, examining a sample of 143 adults aged 65–80 years, suggested that enhancing one’s dietary natural antioxidant intake could play an important role in the prevention of CVDs [26]. Finally, it is confirmed that a high daily consumption of antioxidant nutrients could play a protective role against oxidative stress [27].

As has been shown in previous research, oxidative stress may impact different aspects of cardiovascular health, such as obesity, hypertension, and type II diabetes mellitus [28]; thus, the present results may indicate a new pathway through which public health strategies regarding the prevention of CVDs from childhood and early adolescence could be formed.

### Limitations

When interpreting the outcomes of the present study some limitations are necessary to be considered. First, as an observational study, neither a causal nor a temporal relationship can be drawn. Second, due to the participants’ original residence being limited to mostly urban areas in Greece, the study’s representativeness was assessed and confirmed when the characteristics for included and not-included areas of Greece were shown to be similar; however, generalization of the results at a global level is not suggested. Nevertheless, a stratified random sampling scheme was employed at the school level, and the study sample size was substantial. Additionally, the presence of school educators and qualified investigators during the questionnaire completion process helped to clarify any possible misunderstandings and increased the validity of the replies provided by the students, limiting reporting bias; however, the methods used to assess dietary habits and physical activity relied on the students’ objective and honest answers.

## 5. Conclusions

This is one of the first studies evaluating the association of an antioxidant diet on body weight status in preadolescence. The results indicated a significant inverse association between antioxidant micronutrient intakes and overweight or obesity status, underlying the need for the promotion of dietary patterns rich in antioxidants to facilitate healthy habits early in life, and to fight the obesity threat.

## Figures and Tables

**Table 1:** Characteristics of students by body weight status.

Characteristics	Overall (n = 1580)	Normal Weight (n = 1142)	Overweight/Obesity (n = 438)	*p*
Age (years)	11.21 ± 0.782	11.23 ± 0.782	11.14 ± 0.777	0.044 *
Gender				<0.001 *
Girls	861 (54.5%)	658 (57.6%)	203 (46.3%)	
Boys	719 (45.5%)	484 (42.4%)	235 (53.7%)	
Mean Energy Intake (kcal/day)	1378 ± 719	1388 ± 726	1353 ± 702	0.391
Physical Activity				<0.001 *
Yes	1243 (78.7%)	923 (80.8%)	320 (73.1%)	
No	337 (21.3%)	219 (19.2%)	118 (26.9%)	
Carbohydrate Intake (g/day)	179.6 ± 95.4	181.6 ± 95.4	174.4 ± 95.3	0.182
Protein Intake (g/day)	59.8 ± 36.6	60.1 ± 37.0	59.0 ± 35.4	0.614
Fat Intake (g/day)	46.7 ± 28.1	46.8 ± 28.5	46.8 ± 27.3	0.895
Vitamin A Intake (μg/day)	1028 ± 890	1053 ± 885	963 ± 900	0.072
Vitamin C Intake (mg/day)	227 ± 161	231 ± 160	215 ± 163	0.069
Vitamin E Intake (mg/day)	6.5 ± 5.1	6.6 ± 5.1	6.4 ± 5.2	0.480
Selenium Intake (μg/day)	67 ± 50	68 ± 51	65 ± 49	0.399
Zinc Intake (mg/day)	6.8 ± 3.8	6.9 ± 3.9	6.7 ± 3.9	0.406
Magnesium Intake (mg/day)	284 ± 168	287 ± 170	276 ± 164	0.202

Data are presented as mean ± standard deviation for quantitative variables, and counts (percentages) for categorical variables. The level of significance is set at *p* < 0.05. * Denotes statistically significant associations.

**Table 2:** Characteristics of students by DAI group.

Characteristics	DAI < −1.17 (n = 790)	DAI ≥ −1.17 (n = 790)	*p*
Age (years)	11.25 ± 0.788	11.16 ± 0.772	0.017 *
Gender			0.173
Girls	444 (56.2%)	417 (52.8%)	
Boys	346 (43.8%)	373 (47.2%)	
Mean BMI (kg/m^2^)	19.46 ± 3.47	18.96 ± 3.38	0.004 *
Mean Energy Intake (kcal/day)	962 ± 337	1794 ± 758	<0.001 *
Physical Activity			0.023 *
Yes	603 (76.3%)	640 (81.0%)	
No	187 (23.7%)	150 (19.0%)	
Carbohydrate Intake (g/day)	123.3 ± 45.2	235.9 ± 99.1	<0.001 *
Protein Intake (g/day)	40.0 ± 13.0	79.6 ± 41.4	<0.001 *
Fat Intake (g/day)	34.4 ± 16.8	59.1 ± 31.6	<0.001 *
Vitamin A Intake (μg/day)	522 ± 328	1534 ± 981	<0.001 *
Vitamin C Intake (mg/day)	128 ± 69	326 ± 165	<0.001 *
Vitamin E Intake (mg/day)	3.5 ± 1.4	9.6 ± 5.6	<0.001 *
Selenium Intake (μg/day)	44 ± 18	90 ± 61	<0.001 *
Zinc Intake (mg/day)	4.5 ± 1.4	9.2 ± 4.1	<0.001 *
Magnesium Intake (mg/day)	174 ± 53	394 ± 172	<0.001 *

Data are presented as mean ± standard deviation for quantitative variables, and counts (percentages) for categorical variables. The level of significance is set at *p* < 0.05. * Denotes statistically significant associations.

**Table 3:** Linear regression models for the evaluation of the DAI on students’ BMI.

	DAI	
	B Coefficient	*p*
Model 1	−0.501	0.004 *
Model 2	−0.607	0.004 *
Model 3	−0.494	0.032 *

Model 1 represents an unadjusted analysis. Model 2 represents an analysis adjusted for age, sex, physical activity, and energy intake. Model 3 is Model 2 further adjusted for intakes of vitamins A, C, and E, selenium, zinc, and magnesium. The level of significance is set at *p* < 0.05. * Denotes statistically significant associations.

**Table 4:** Logistic regression models for the evaluation of the DAI on students’ probability of being of increased weight (overweight/obese).

	DAI	
	Odds Ratio	95% Confidence Interval
Model 1	0.719	(0.576; 0.897) *
Model 2	0.662	(0.502; 0.873) *
Model 3	0.666	(0.489; 0.907) *

Model 1 represents an unadjusted analysis. Model 2 represents an analysis adjusted for age, sex, physical activity, and energy intake. Model 3 is Model 2 further adjusted for intakes of vitamins A, C, and E, selenium, zinc, and magnesium. The level of significance is set at *p* < 0.05. * Denotes statistically significant associations.

## Data Availability

Data available upon request.

## References

[1] World Health Organization: WHO Obesity and Overweight. https://www.who.int/news-room/fact-sheets/detail/obesity-and-overweight#cms.

[2] Simmonds M., Llewellyn A., Owen C.G., Woolacott N. (2015). Predicting adult obesity from childhood obesity: A systematic review and meta-analysis. Obes. Rev..

[3] Hassapidou M. (2022). Prevalence of childhood obesity in Greece: Results from WHO Childhood Obesity Surveillance Initiative 2010–2020. Public Health Toxicol..

[4] Codoñer-Franch P., Valls-Bellés V., Arilla-Codoñer Á., Alonso-Iglesias E. (2011). Oxidant mechanisms in childhood obesity: The link between inflammation and oxidative stress. Transl. Res..

[5] Fernández-Sánchez A., Madrigal-Santillán E., Bautista M., Esquivel-Soto J., Morales-González Á., Esquivel-Chirino C., Durante-Montiel I., Sánchez-Rivera G., Valadéz-Vega C., Morales-González J.A. (2011). Inflammation, oxidative stress, and obesity. Int. J. Mol. Sci..

[6] Vincent H.K., Innes K.E., Vincent K.R. (2007). Oxidative stress and potential interventions to reduce oxidative stress in overweight and obesity. Diabetes Obes. Metab. Diabetes Obes. Metab..

[7] Bondia-Pons I., Ryan L., Martínéz J.A. (2012). Oxidative stress and inflammation interactions in human obesity. J. Physiol. Biochem..

[8] Marseglia L., Manti S., Nicotera A.G., Parisi E., Di Rosa G., Gitto E., Arrigo T. (2014). Oxidative stress in obesity: A critical component in human diseases. Int. J. Mol. Sci..

[9] Manna P., Jain S.K. (2015). Obesity, oxidative stress, adipose tissue dysfunction, and the associated health risks: Causes and therapeutic strategies. Metab. Syndr. Relat. Disord..

[10] Tobore T.O. (2020). Towards a comprehensive theory of obesity and a healthy diet: The causal role of oxidative stress in food addiction and obesity. Behav. Brain Res..

[11] Pérez-Torres I., Castrejón-Téllez V., Soto M.E., Rubio-Ruiz M.E., Manzano-Pech L., Guarner-Lans V. (2021). Oxidative stress, plant natural antioxidants, and obesity. Int. J. Mol. Sci..

[12] Hosseini B., Saedisomeolia A., Allman-Farinelli M. (2016). Association Between Antioxidant Intake/Status and Obesity: A Systematic Review of Observational Studies. Biol. Trace Elem. Res..

[13] Cole T., Bellizzi M., Flegal K.M., Dietz W.H. (2000). Establishing a standard definition for child overweight and obesity worldwide: International survey. BMJ. Br. Med. J..

[14] Antonogeogros G., Grigoropoulou D., Papadimitriou A., Priftis K.Ν., Anthracopoulos M.B., Nicolaidou P., Panagiotakos D.B. (2011). Validation of a food frequency questionnaire designed for children 10-12 years: The Panacea-FFQ. Pediatr. Res..

[15] Argiropoulou E.C., Michalopoulou M., Aggeloussis N., Avgerinos A. (2004). Validity and reliability of physical activity measures in greek high school age children. J. Sports Sci. Med..

[16] FoodData Central. https://fdc.nal.usda.gov/fdc-app.html#/.

[17] Τrichopoulou A., Georga K. (2004). Composition Tables for Greek Foods and Recipes.

[18] Wright M.E., Mayne S.T., Stolzenberg-Solomon R.Z., Li Z., Pietinen P., Taylor P.R., Virtamo J., Albanês D. (2004). Development of a comprehensive dietary antioxidant index and application to lung cancer risk in a cohort of male smokers. Am. J. Epidemiol..

[19] Aminnejad B., Roumi Z., Ardekanizadeh N.H., Vahid F., Gholamalizadeh M., Kalantari N., Ataei A., Doaei S. (2022). Association of dietary antioxidant index with body mass index in adolescents. Obes. Sci. Pract..

[20] Vahid F., Rahmani D., Davoodi S.H. (2020). Validation of Dietary Antioxidant Index (DAI) and investigating the relationship between DAI and the odds of gastric cancer. Nutr. Metab..

[21] Mohammadi S., Lotfi K., Mirzaei S., Asadi A., Akhlaghi M., Saneei P. (2022). Dietary total antioxidant capacity in relation to metabolic health status in overweight and obese adolescents. Nutr. J..

[22] Azizi-Soleiman F., Khoshhali M., Heidari-Beni M., Qorbani M., Kelishadi R. (2020). Association between Dietary Antioxidant Quality Score and Anthropometric Measurements in Children and Adolescents: The Weight Disorders Survey of the CASPIAN-IV Study. J. Trop. Pediatr..

[23] Puchau B., Ochoa M.C., Zulet M.Á., Marti A., Martínéz J.A., Members G. (2010). Dietary total antioxidant capacity and obesity in children and adolescents. Int. J. Food Sci. Nutr..

[24] Wang W., Wang X., Cao S., Duan Y., Xu C., Gan D., He W. (2022). Dietary Antioxidant Indices in Relation to All-Cause and Cause-Specific Mortality among Adults with Diabetes: A Prospective Cohort study. Front. Nutr..

[25] Vahid F., Rahmani D., Davoodi S.H. (2021). The correlation between serum inflammatory, antioxidant, glucose handling biomarkers, and Dietary Antioxidant Index (DAI) and the role of DAI in obesity/overweight causation: Population-based case–control study. Int. J. Obes..

[26] Kolarzyk E., Skop-Lewandowska A., Jaworska J., Ostachowska-Gąsior A., Krzeszowska-Rosiek T. (2018). Dietary intake of antioxidants and fats in the context of coronary heart disease prevention among elderly people. AAEM Ann. Agric. Environ. Med. Ann. Agric. Environ. Med..

[27] Kolarzyk E., Pietrzycka A., Zając J., Morawiecka-Baranek J. (2017). Relationship between dietary antioxidant index (DAI) and antioxidants level in plasma of Kraków inhabitants. Adv. Clin. Exp. Med..

[28] Hertiš Petek T., Petek T., Močnik M., Marčun Varda N. (2022). Systemic Inflammation, Oxidative Stress and Cardiovascular Health in Children and Adolescents: A Systematic Review. Antioxidants.

